# Primary care healthcare policy implementation in the Eastern Mediterranean region; experiences of six countries: Part II

**DOI:** 10.1080/13814788.2019.1640210

**Published:** 2019-08-01

**Authors:** Nagwa Nashat, Redouane Hadjij, Abdul Munem Al Dabbagh, Mohammed Rasoul Tarawneh, Huda Alduwaisan, Fatima Zohra, Eiad Abdelmohsen AlFaris, Harumi Quezada-Yamamoto, Chris van Weel, Salman Rawaf

**Affiliations:** a Department of Family Medicine, Menoufia University, Menoufia, Egypt;; b Algerian Society of General Medicine, Alger, Algeria;; c Iraqi Board for Medical Specializations, Scientific Council of Family and Community Medicine, Baghdad, Iraq;; d Jordan Society of Family Medicine, Secretary General High Health Council, Amman, Jordan;; e Kuwait Society for Family and General Practitioners, Kuwait City, Kuwait;; f MG MAROC, Moroccan General Practitioner Association, Rabat, Morocco;; g King Saud University Chair for Medical Education Research and Development, Department of Family and Community Medicine, College of Medicine, King Saud University, Riyadh, Saudi Arabia;; h UK: Imperial College London & WHO Collaborating Centre, London, UK;; i The Netherlands Radboud Institute of Health Sciences, Department of Primary and Community Care, Radboud University Medical Centre, Nijmegen, The Netherlands;; j Department of Health Services Research and Policy, Australian National University, Canberra, Australia

**Keywords:** Primary healthcare, community health services, healthcare facilities, healthcare quality, access

## Abstract

**Background:** Primary healthcare (PHC) is essential for equitable access and cost-effective healthcare. This makes PHC a key factor in the global strategy for universal health coverage (UHC). Implementing PHC requires an understanding of the health system under prevailing circumstances, but for most countries, no data are available.

**Objectives:** This paper describes and analyses the health systems of Algeria, Kuwait, Morocco, Saudi Arabia, Jordan and Iraq to PHC.

**Methods:** Data were collected during a workshop at the Wonca East Mediterranean Regional Conference in 2018. Academic family physicians (FP) presented their country; using the Wonca framework of 11 PowerPoint slides, with queries of the country demographics, main health challenges, and the position of PHC in the health system.

**Results:** The six countries had achieved a significant improvement in populations’ health but currently face challenges of health financing, a small number of certified FPs, difficulties in accessing services and bureaucratic procedures. Primary concerns were the absence of a family practice model, brain drain and immigration of FPs. Countries differed in building a coherent policy.

**Conclusion:** Priorities should be focused on: developing PHC model in Eastern Mediterranean region with advocacy for community-based PHC to policymakers; capacity building for strengthening PHC-oriented health systems with FP specialty training and restrict practising to fully trained FPs; engage communities to improve understanding of PHC; adopt quality and accreditation policies for better services; validation of the referral and follow-up process; and, develop public–private partnership mechanisms to enhance PHC for UHC.

KEY MESSAGES

- Some Eastern Mediterranean countries lack a family medicine model and training programme, while others need upscaling.
- Primary care should be proactive, moving away from disease-based and towards a comprehensive health-for-all model, through integrated and continuous service delivery.
- Policymakers should commit the resources needed for family medicine.

## Introduction

This paper is a continuation of the previous publication under the same name, now analysing six other countries after the 2018 Wonca East Mediterranean Regional Conference. The country members of Wonca East Mediterranean Region are Saudi Arabia, Lebanon, Bahrain, Jordan, Iraq, United Arab Emirates (UAE), Oman, Egypt, Syria, Qatar, Morocco, Algeria, Afghanistan, Kuwait and Iran.

The Astana Declaration 2018 reaffirmed Alma-Ata’s principle that primary healthcare (PHC) is the most inclusive, effective and efficient approach to enhance people’s physical and mental health, as well as social wellbeing, and that PHC is a cornerstone of a sustainable health system for universal health coverage (UHC) and health-related sustainable development goals [1]. One of the most effective and valuable principles to promote health in all countries has been the adoption of PHC as a fundamental strategy for justice in the delivery and distribution of services in the health sector, paving the way towards the achievement of UHC [1–3]. Insight into countries’ health systems is important to plan effective ways to strengthen health systems. Currently, there is vast knowledge and experience available globally on how to implement PHC policy under prevailing social and economic conditions [4–8]. However, there is a limited insight into the value of PHC in many other countries. To address this gap, the Wonca Working Party on Research decided to document PHC around the world, and stimulate dialogues of how the values of PHC can be re-addressed within the constraints of different health systems [9]. Earlier studies documented the Asia-Pacific, South Asia regions, Mexico and some Eastern Mediterranean region (EMR) countries [10–12].

This paper seeks to continue and complete documentation and critical appraisal of the health systems of the EMR, by reviewing the six countries not yet analysed: Algeria, Iraq, Jordan, Kuwait, Morocco and Saudi Arabia; with the objective of identifying common strategies for strengthening PHC, prioritizing regional collaboration and exploring collaboration with the World Health Organization (WHO) Regional Office for EMR.

## Methods

A workshop at the March 2018 Wonca EMR Conference in Kuwait, following the same outline as the first workshop at the 2017 in Abu Dhabi [12], compared the health systems of the other six Wonca member countries: Algeria, Iraq, Jordan, Kuwait, Morocco, and Saudi Arabia, from which the data for this paper was provided. Although Algeria is not part of the EMR Office of WHO, as a Middle Eastern Arab country, it was logical to include it within this group of countries.

In this paper, family physicians (FP) are referred to as PHC-specialty trained physicians and general practitioners (GPs) as those without further specialization training after graduating from medical school.

An academic FP from each country was selected to present their country, using the Wonca framework of 11 PowerPoint slides that focused on country demographics, the health system and the position of PHC, the country’s leading health challenges, strengths and weaknesses and lessons others could learn from [9]. They were free to concentrate on what, in their view, were the most critical aspects. All workshop presenters and moderators contributed to the discussion, directed to strategies to strengthen PHC and the contribution that regional and international collaboration could make. Presentations, discussions and summaries formed the basis of this article.

## Results

### Country profiles

Algeria is an upper middle-income country, with a population of 40 million, and served by 13.2 physicians per 10 000 inhabitants, working in both the public and private sectors [13]. The public sector represents 60% of the volume of service delivery, with 30% of the total number of physicians. The insurance funds reimburse prescribed medication for 80% of the population. There are 24 831 GPs distributed in the public sector, while there are 9000 in private sector clinics [14,15]. Most physicians practice in the state-funded public sector, working in well-structured care teams (nurses, laboratory technicians, pharmacists, dentists). In the public health system, the GP deals with 90% of the population at first contact. The rest of the patients are referred to health centres of universities and emergency health services. The lack of a structured training programme for family medicine is a major challenge in Algeria.

Iraq is an upper middle-income country that has 40 million inhabitants, served by 7.4 physicians per population of 10 000 [13]. PHC services are well provided with 2669 centres distributed all over the country. Of these centres, 1259 are ‘main PHCCs’ and 1410 are health units. Currently, 119 centres apply the family medicine model. Each main PHCC is run by certified FPs, GPs and other specialities like internal medicine, paediatrics and gynaecology. The density of FPs is less than 1.45 per 10 000 population. These PHC centres provide wide range of services including health promotion, preventive, and curative health services. The health units are operated by a GP or a medical assistant providing essential medical services and basic emergency care. PHC financing, shortage of trained and certified FPs, brain drain and migration due to the exceptional situation in Iraq are the main barriers towards achieving quality PHC. The country is currently increasing training capacity through a national Iraqi board and a regional Arab board. However, UHC may be remote in this country unless the challenges mentioned above are adequately addressed [16,17].

Jordan is a lower middle-income country that occupies an area of 89 342 km^2^ and with a population of nearly 10 million, 30% of which are migrants or refugees from neighbouring countries [13]. There are 2.26 physicians per population of 1000 [18]. The PHC team consists of FPs, GPs, nurses, assistant nurses, midwives, social workers, pharmacists and other allied health professionals. Less than 10% of primary care doctors are fully trained FPs. There are three levels of health centre: PHC, comprehensive health centre, and village health centre. Each of the 380 PHCs headed by a GP, provides curative and preventive healthcare including dental and schoolchildren services. The 102 comprehensive health centres provide speciality care in the areas of paediatrics, gynaecology, internal medicine, orthopaedics, ENT, ophthalmology, dermatology and dentistry. The 194 village health centres provide health promotion in villages and maintain simple information on vital statistics (births, deaths, etc.). Other providers of PHC are the Royal Medical Service (12 PHCC), universities (four centres), and United Nations Relief and Work Agency for Palestine Refugees (UNRWA) (25 centres). Furthermore, the Ministry of Health provides 464 maternity and child centres and 12 chest disease centres classified as part of primary care service. Community health committees, which consist of community volunteers, identify the local needs through different activities and communicate with HC team. These Committees represent members and local non-governmental organizations (NGOs) in addition to members of the health centres, where they regularly meet to discuss and implement plans to address local issues. Challenges encountered include insufficiently trained workforce specifically FPs, lack of systematic approach to the community-based activities, and lack of incentives for community volunteers [19]. Government plans to expand family medicine training. However, resources are not identified [20].

Kuwait is a high-income country with a population of 4.1 million; 70% of which are expatriates [13]. There are 1.8 physicians per population of 10 000; about one third are working in the private sector, representing 20% of the total health expenditure. The Ministry of Health is responsible for PHC and part of the public service. Recently, health insurance was introduced for retired nationals and with marginal cover for expatriates. There are 103 PHC centres, with one-third of primary care doctors certified as FPs (410 in 2018). These centres provide essential health service packages (EHSP) and interventions that are based on community priorities and needs. There is no fixed or ideal number of staff for all PHC facilities, as the population coverage and type of services needed to be delivered may vary from one centre to another. Half the PHCs (50%) have laboratory and X-ray services, dental clinics, geriatric clinics, and obstetrics and gynaecology units. Community-based PHC in Kuwait is a collaboration between PHC centres and other community services, in forms of specific initiatives. Community leaders support PHC centres in the form of sponsorships and active participation in initiatives. There is no solid structure or policy for such interaction. PHC teams are empowering communities to identify their own needs and provide solutions for them. Through District Community Councils, needs are assessed, and services are provided to achieve the targeted goals [21]. Kuwait has a national board for training in family medicine.

Morocco is a lower middle-income country with 34 million people over an area of 710 850 km^2^ and has 0.68 physicians per population of 10 000 [13]. The public sector, Royal Armed Forces, the private sector, and informal sectors provide PHC services. The public sector is responsible for two levels of delivery. The first level of health centres, under the responsibility of a GP and a nurse, provides health promotion, preventive, and curative care. The second level health centres are composed of emergency, oral, and mental health services. The private sector provides PHC service through ‘medical practices’, which are run either by an individual or group of GPs. In general, Morocco has advanced its health system in addressing inequities in service provision. The advent of mandatory medical coverage (‘AMO’ and ‘Ramed’) has been a real social evolution for Moroccans. This will obligate the state to implement the regulation of healthcare providers and control costs in the private and public sectors. However, many challenges are encountered, such as difficulties in accessing services due to geographical concentration and both qualitative and quantitative deficit in health professionals, as no specialized training for family medicine is available [22,23]. The two-year master’s degree programme in Family and Community Health developed in 2015 is not a substitution for the urgent need to scale up family medicine training.

Saudi Arabia is a high-income country with a population of 33 million: 33% of which are non-nationals [13]. There are 2.5 physicians per population of 10 000. Out of 6107 primary care doctors, 636 were fully trained in 2018. The Saudi healthcare system was ranked 26 among 190 countries and its total expenditure on health in 2014 was 4.7% of the gross domestic product [24,25]. Despite many efforts by the Saudi government to meet the healthcare needs, challenges still exist. These include increasing cost of healthcare exerting financial pressure on the Saudi healthcare system, high population growth rate, high life expectancy, healthcare provision for pilgrims (Hajj), under-resourced primary care, and underutilization of resources in tertiary care due to lack of accountability, governance and coordination between different institutes [26]. Only 11 million in 2015 of the Saudi population were insured by 2015. The number of PHC centres increased from 1800 in 2004 to 2390 in 2018. Currently, the Ministry of Health is the major governmental provider, regulatory authority, and financer of healthcare services, which run 60% of the health services. Other governmental sectors represent 13% and the private sector represents 27% (26). In 2016, ‘Saudi Arabia Vision 2030,’ a wide-ranging privatization and economic reform programme was introduced, including the National Transformation Programme 2020. This requires each public institute to improve its performance through cooperation and partnership with the private sector [27]. The Ministry of Health produced new initiatives aiming to reduce costs through sharing risks between public and private institutions and engaging community-driven approaches within the healthcare system [28,29]. The initiatives included reform and restructuring of PHC, the establishment of private–public partnerships, and the privatization of one of the medical complexes also called ‘cities.’ The target for 2020 is increasing private healthcare expenditure to 35%.

## Discussion

Each of the countries presented had a unique situation but all had PHC on their agenda. Four ‘themes’ emerged from the above analysis of the six countries. There is a need:

- for a distinct description of the roles of the FP and GP, the responsibilities of such, and career pathways;
- for leadership and governance;
- for multi-sectorial collaboration; and
- for allocation and management of financial resources.

As with the previous study [12], policymakers must address these common challenges.

### Implications: Distinct description of the family physician

It was well noted in the previous study that all countries studied had an implemented family practice system [12]. However, this study showed that Maghreb countries lack the concept of family medicine in their health system. Policymakers must work towards a career pathway to ensure that FPs play their crucial role in improving population health and achieve UHC. A similar finding was observed throughout the entire EMR region. It was well known that the number of certified FPs is very small. In addition, the disease-based model of service, difficulties with access to service along with the negative public perception of PHC and bureaucratic procedures are the main characteristics. Overall, the main challenges were the absence of a family practice model in some countries, with brain drain and immigration of FPs being the challenging factors in others. Countries differed in the extent to which this had resulted in a coherent policy.

### Implications: Towards the WHO Eastern Mediterranean (EMRO) assessment strategy

The analysis also showed that all countries analysed must upscale training of FPs to meet the population health service needs. All of these countries have less than three FPs per 10 000; a standard recommended by WHO EMRO [30]. Without this progression, UHC will not be achieved and many people will be left without care. In line with sustainable development goals (SDGs), achieving UHC may even require a much higher number of fully trained FPs and radically reshape primary care to meet the modern population’s health needs [31].

According to the WHO assessment strategy to measure the level of PHC implementation, 13 elements are required (Box 1). Related to this is the importance of policymakers recognizing the need to lead PHC through specialists in that domain. Policy on FP specialization should be accompanied by health regulations that restrict the possibility of practice in the PHC setting to FP certification, and provide FPs access to a broad and comprehensive set of diagnostic and therapeutic interventions to respond to the prevalent health problems in the community.

Box 1 Elements required to measure PHC implementation

1. Government commitment
2. Registration of catchment population and development of family folder
3. Development of family roster
4. Community engagement and inter-sectorial action
5. Essential health services package
6. Essential medicines list
7. Staff pattern based on family practice with updated job descriptions
8. Standard set of medical equipment and furniture
9. Training of FPs and short-term, on-the-job training for GPs
10. Treatment protocols
11. Referral system
12. Information system for family practice and quality
13. Safety and accreditation programme

The Astana Declaration, 2018 gave clear directions to WHO member states on how to achieve UHC through PHC and develop models that integrate public health and primary care at first contact with the health system to achieve better health for all, leaving no one behind [1,32]. WHO EMRO and member states need to examine the best evidence policy accelerators for transforming primary care in EMR [33]. Primary care saves lives and improves health.

## Conclusion

In the described countries, priorities should be focused on (i) developing a successive PHC model in all the EMR with policymaker advocacy for community-based PHC, (ii) assisting capacity building at country level for strengthening PHC-oriented health systems; (iii) engaging communities to improve public understanding of PHC; (iv) adopting quality and accreditation policies to improve the quality of PHC services; (v) validating referral and follow up processes; and (vi) developing public–private partnership mechanisms to focus on PHC and UHC.
